# Primary Human Ovarian Epithelial Cancer Cells Broadly Express HER2 at Immunologically-Detectable Levels

**DOI:** 10.1371/journal.pone.0049829

**Published:** 2012-11-26

**Authors:** Evripidis Lanitis, Denarda Dangaj, Ian S. Hagemann, De-Gang Song, Andrew Best, Raphael Sandaltzopoulos, George Coukos, Daniel J. Powell

**Affiliations:** 1 Ovarian Cancer Research Center, Department of Obstetrics and Gynecology, University of Pennsylvania, Philadelphia, Pennsylvania, United States of America; 2 Department of Molecular Biology and Genetics, Democritus University of Thrace, Alexandroupolis, Greece; 3 Abramson Cancer Center, Department of Pathology and Laboratory Medicine, University of Pennsylvania, Philadelphia, Pennsylvania, United States of America; Baylor College of Medicine, United States of America

## Abstract

The breadth of HER2 expression by primary human ovarian cancers remains controversial, which questions its suitability as a universal antigen in this malignancy. To address these issues, we performed extensive HER2 expression analysis on a wide panel of primary tumors as well as established and short-term human ovarian cancer cell lines. Conventional immunohistochemical (IHC) analysis of multiple tumor sites in 50 cases of high-grade ovarian serous carcinomas revealed HER2 overexpression in 29% of evaluated sites. However, more sensitive detection methods including flow cytometry, western blot analysis and q-PCR revealed HER2 expression in all fresh tumor cells derived from primary ascites or solid tumors as well as all established and short-term cultured cancer cell lines. Cancer cells generally expressed HER2 at higher levels than that found in normal ovarian surface epithelial (OSE) cells. Accordingly, genetically-engineered human T cells expressing an HER2-specific chimeric antigen receptor (CAR) recognized and reacted against all established or primary ovarian cancer cells tested with minimal or no reactivity against normal OSE cells. In conclusion, all human ovarian cancers express immunologically-detectable levels of HER2, indicating that IHC measurement underestimates the true frequency of HER2-expressing ovarian cancers and may limit patient access to otherwise clinically meaningful HER2-targeted therapies.

## Introduction

The *ERBB2* proto-oncogene encodes a transmembrane protein tyrosine kinase receptor involved in the development and progression of many cancers including ovarian cancer [1], [2]. Dysregulated HER2 signaling in ovarian cancer (OvCa) results from either gene amplification or overexpression and leads to faster cell growth [3], improved DNA repair [4] and increased colony formation [5]. HER2 overexpression is associated with an increased risk of progression and death especially among women with FIGO stage I and II OvCa [6]. However, no correlation has been found between the presence of HER2 overexpression and FIGO stage, suggesting that activation of HER2 overexpression is broad and can occur both in early and late stages of disease [7]. These qualities would appear to make HER2 an attractive molecule for targeted immunotherapies in women with HER2-positive ovarian cancer, where naturally-occurring CD4^+^ and CD8^+^ T cell responses have been observed [8].

HER2 protein expression is most commonly detected via semi-quantitative IHC analysis on paraffin embedded tissues using established protocols employed for the assessment of breast cancer patients being considered for anti-HER2 Herceptin (trastuzumab) treatment [9]. The extent to which HER2 is expressed by OvCas remains controversial, as the rate of HER2-positive OvCas reported in the literature ranges from 4.9% to 52.5% [6], [7], [10], [11], [12], [13], [14], [15]. However, in a single study performed by Hellstrom et al., all tumor cell lines that were established *in vitro* from solid tumor or ascites expressed HER2 suggesting a selective growth advantage for HER2-positive cancer cells in culture [16]. One established cell line was shown to be sensitive to HER2-directed antibody-dependent cellular cytotoxicity (ADCC), however, HER2 expression and ADCC sensitivity was not assessed on cells derived from physiological ovaries. Additionally, HER2 expression analysis utilized flow cytometry as the sole detection method and was limited to a relatively small number of cases, relying heavily upon in vitro cell culture.

In the current study, established ovarian cancer cell lines, primary short-term cultured cell lines and fresh ovarian cancer cells derived from ascites and solid tumor specimens were evaluated for HER2 expression utilizing various detection methods, including quantitative PCR (q-PCR), western blot analysis and flow cytometry, and expression levels were compared to corresponding levels in normal ovarian surface epithelium cells. Further, immunologically-active levels of HER2 were measured using human T cells that were genetically engineered to express an HER2-specific chimeric antigen receptor (CAR). Anti-HER2 CAR T cells were evaluated for their capacity to recognize HER2-expressing OvCas and normal cells. Our results demonstrate that all OvCa samples express HER2, and that this level of expression is sufficient to elicit immune recognition.

**Figure 1:**
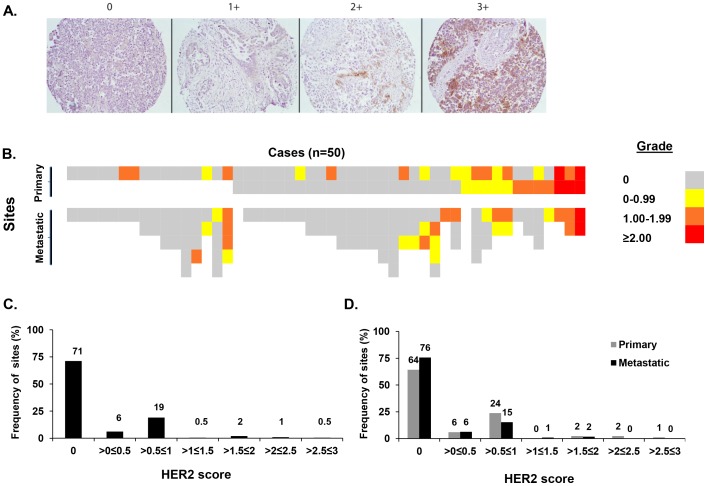
Immunohistochemical detection of HER2 in ovarian cancer. ***A.*** Immunostaining showing regional diversity of HER2 expression in high-grade papillary serous ovarian adenocarcinoma. HER2 expression levels were graded in a 0–3 scale (score 0 =  undetectable, score 3 =  strong staining). Original magnification was 200×. ***B.*** Heatmap illustration of HER2 expression level in 50 primary and metastatic ovarian carcinoma cases, as scored by IHC staining. ***C.*** Frequency distribution of sites expressing HER2 at levels ranging from score 0 to 3. The mean number of evaluated sites for cases with undetectable or detectable HER2 expression was similar (4.2 vs. 3.7 respectively; *P* = 0.19). ***D.*** Frequency distribution of either primary or metastatic sites expressing HER2 at levels ranging from score 0 to 3. A greater frequency of primary tumor sites expressed HER2 (36%; 30/84) compared to metastatic sites (24%; 27/111) and had a higher mean HER2 expression score (0.37 *vs.* 0.21, *P* = 0.04). No statistically significant difference was observed comparing the expression levels among the primary and metastatic sites that expressed HER2 at any level (1.03 *vs.* 0.86, *P* = 0.26). *P* values were calculated using unpaired student’s t-test analysis.

## Materials and Methods

### Cancer Cells and Lines

Donors entered into a University of Pennsylvania Institutional Review Board (IRB)-approved clinical protocol and signed an informed consent prior to tumor or blood collection. For solid tumors or normal ovarian samples, specimen was diced in RPMI-1640, washed and centrifuged (800 rpm, 5 minutes, 15–22°C), and resuspended in enzymatic digestion buffer (0.2 mg/ml collagenase and 30units/ml DNase in RPMI-1640) for overnight rotation at room temperature. Ascites collections were washed and cryopreserved before study. Short-term cultured primary lines were kindly provided by Dr. Richard Carroll at the University of Pennsylvania [17]. Established human ovarian and breast cancer cell lines, the CEM human T cell lymphoblast-like cell line and the 293T cell line were purchased (ATCC). Normal IOSE-4 and IOSE-6 cell lines were kindly provided by Dr. Birrer from Dana-Farber/Harvard Cancer Center [18] and the 398 cell line was a gift from Dr. Lin Zhang at the University of Pennsylvania [19]. 293T cells and tumor cell lines were maintained in complete medium; RPMI-1640 (Invitrogen) supplemented with 10% (v/v) heat-inactivated FBS, 2 mM L-glutamine, and 100 µg/mL penicillin and 100 U/mL streptomycin.

**Figure 2:**
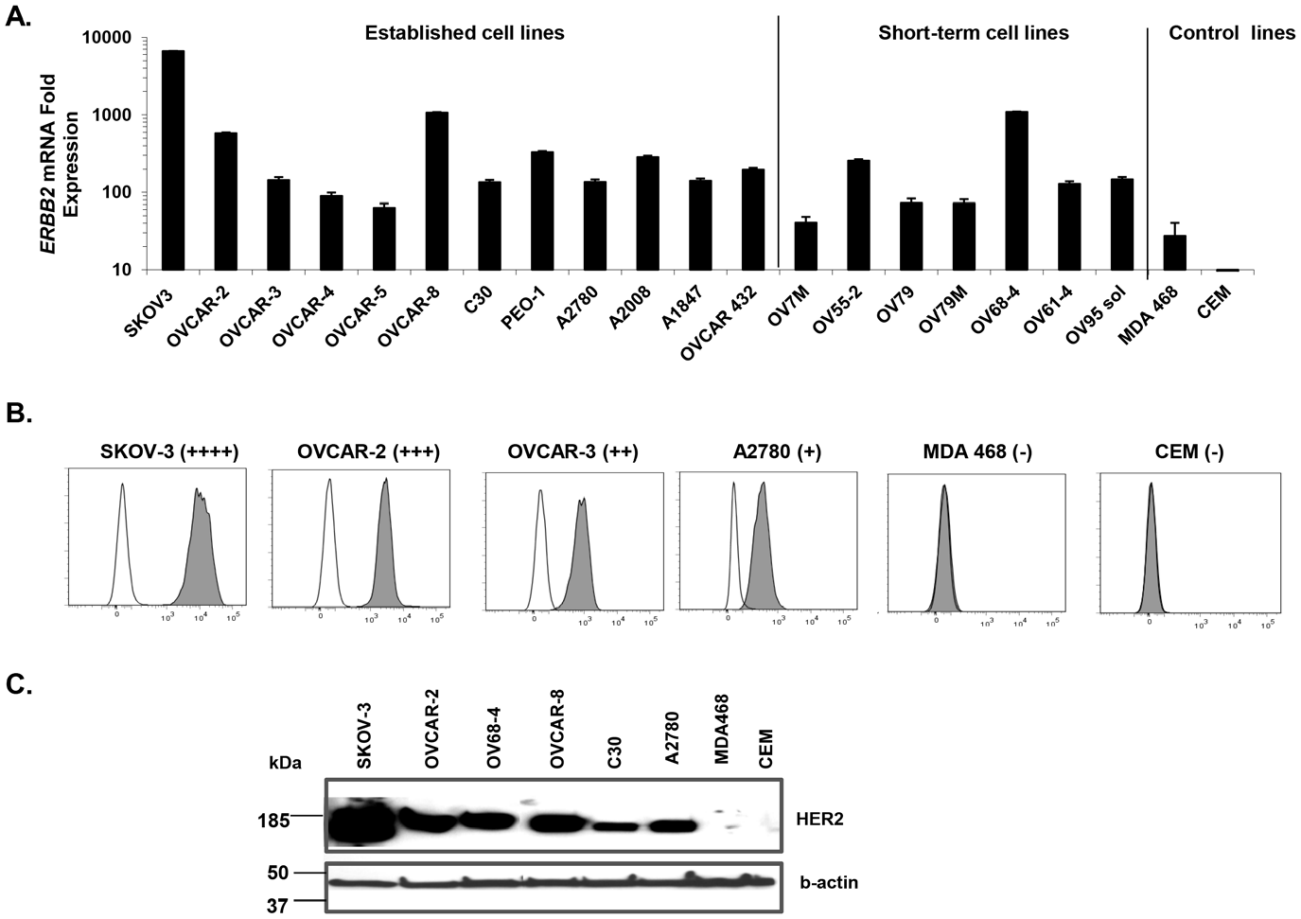
Ubiquitous HER2 expression in ovarian cancer cell lines. ***A.*** *ERBB2* mRNA levels in ovarian cancer cell lines by q-PCR. *ERBB2* mRNA levels of ovarian cancer cell lines are relative to that of ErbB2-negative CEM cells. All ovarian cancer cell lines express *ERBB2* mRNA. B-actin was used as an endogenous gene control. Results depict the mean ± SD of triplicate wells. Mean relative *ERBB2* mRNA expression amongst established and short-term OvCa cell lines was not statistically different (*P* = 0.45). *P* value was calculated using unpaired student’s t-test analysis. ***B.*** Detection of surface HER2 protein expression (filled histograms) by human ovarian cancer cell lines by flow cytometry; isotype antibody control (open histograms). ***C.*** Western blot analysis of HER2 protein expression in representative cell lines expressing differential amounts of HER2. HER2 protein is expressed at variable levels in all the ovarian cell lines tested. B-actin was used as control.

### Immunohistochemistry

Institutional review board approval was obtained. We retrieved records from 50 consecutive patients with metastatic papillary serous ovarian cancer (FIGO stage IIB and above) undergoing primary resection at our institution between 2005 and 2008. Slides were reviewed and annotated and paraffin-embedded tissue blocks were selected to construct a tissue microarray of primary and metastatic tumors. 206 total tumor deposits (primary sites and metastases) were represented on the array. A mean of 3.7 sites were included per patient. The most common metastatic sites included omentum, peritoneum (e.g., cul-de-sac), uterine serosa, and bowel wall. For each block, triplicate 0.6 mm cores of tumor were placed on a tissue microarray. 5 µm paraffin sections were stained with rabbit anti-human HER2 antibody (Dako) according to standard protocols. HER2 expression in each core was scored by light microscopy at 200× magnification using a semiquantitative scale ranging from 0 to 3. Cores showing less than 10% tumor were not scored. For each tumor site, the final score was the mean of the scores for all evaluable cores.

**Table 1:** HER2 surface protein expression in established and primary ovarian cell lines.

Established ovarian cell lines			Short-term ovarian cell lines		
Tumor ID	Specific HER2 MFI	% HER2 positive cells	Tumor ID	Specific HER2 MFI	% HER2 positive cells
SKOV-3	15123	99.7	OV68-4	1261	99.6
OVCAR-2	2946	100	OV55-2	1130	84
OVCAR-3	932	99.6	OV61-4	644	98.8
PEO-1	726	99.9	OV7M	367	96.9
OVCAR-8	616	99.8	OV79	298	94.7
A2008	604	98.9	OV95 Sol	270	83.3
A1847	563	97.5	OV79M	190	64.7
OVCAR-4	475	99	Negative control cell lines		
OVCAR-5	275	98.6	MDA 468	8	1
OVCAR 432	235	98.9	CEM	8	1.9
C30	210	96.1			
A2780	161	84.1			

HER2 is expressed in the cell surface of all ascites and solid tumor-derived tumor cells. HER2 expression was assessed using flow cytometry in a large panel of clinical ascites and solid tumor specimens. A breast cancer cell line (MDA 468) and a leukemia cell line (CEM) which do not express any HER2 were used as negative controls. HER2 was found to be expressed in all tested ascites and solid tumor specimens albeit at different levels. HER2 cell surface levels are expressed as specific MFI. *P* = 0.03 when comparing percentage of HER2 protein-expressing cells in established vs. short-term lines. *P* = 0.43 when comparing MFI of HER2 protein detection in established vs. to short-term cell lines. *P* values were calculated using student’s unpaired t-test analysis.

### Quantitative PCR

RNA was isolated using RNA easy kit (Qiagen). cDNA was generated from 1 ug of RNA using First Strand Ready-To-Go beads (GE Healthcare). Real-time PCR was performed in triplicates using Applied Biosystem’s primers for *ERBB2* and B-actin. *ERBB2* mRNA levels in cancer cells were normalized to B-actin, and compared to those in CEM, and are presented as fold *ERBB2* mRNA level. Data acquisition and analysis was performed according Applied Biosystem’s instructions.

**Figure 3:**
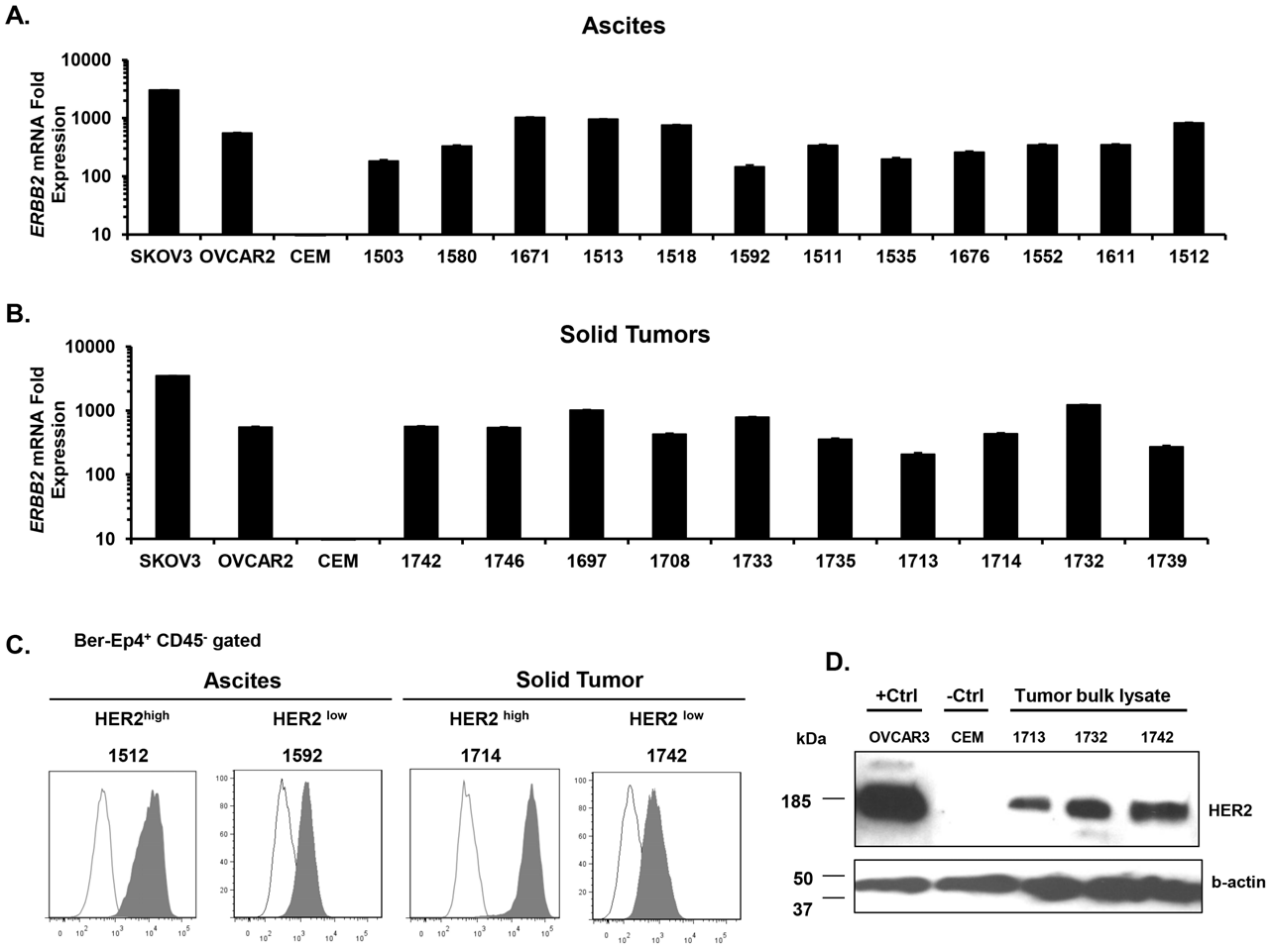
All primary cancers express HER2 protein and ***ERBB2*** **mRNA.** ***A*** **.** *ERBB2* mRNA quantitation in CD45-depleted primary ascites cancer cells. SKOV-3 and CEM were used as positive and negative HER2-expressing cell line controls respectively. Bars depict the mean ± SD values of triplicate wells. ***B*** **.** *ERBB2* mRNA quantitation in CD45-depleted primary solid tumor cells. No significant difference in mean *ERBB2* mRNA level was observed between ascites and solid tumors (*P* = 0.46).***C.*** Surface HER2 expression (solid histograms) by representative Ber-Ep4^+^ CD45^−^ gated ascites and solid tumor-derived cancer cells monitored by flow cytometry; isotype antibody control (open histograms). No significant difference in HER2 protein level was observed between ascites or solid tumors derived cells (*P* = 0.95). ***D.*** Western blot analysis of HER2 protein expression in bulk solid tumor lysates. B-actin was used as endogenous gene control. OVCAR-3 and CEM were used as positive and negative controls for HER2 expression. P values were calculated using unpaired student’s t-test analysis.

**Table 2:** HER2 surface protein expression in fresh primary ovarian ascites, solid tumor and normal OSE.

Primary Ascites			Solid Tumors		
Tumor ID	Specific HER2 MFI	% HER2 positive cells	Tumor ID	Specific HER2 MFI	% HER2 positive cells
734	23566	92	1714	22760	98.2
1512	15510	85.8	1735	17824	96.9
1680	13570	76.6	1708	15084	97.7
1565	13099	69.2	1733	4445	72.6
1665	13000	86.4	1697	4318	50.3
1511	10917	93.7	1713	3805	55.3
1671	8769	87.5	1746	2023	59
1515	7359	72.7	1732	1726	64.5
1518	6911	88.7	1742	1044	17.5
1513	6094	91.1	1739	1041	57.8
1659	5128	69.5	Control cell lines		
1611	4776	73.3	SKOV-3	22935	100
1552	4210	67.6	OVCAR-2	7206	100
1676	3996	64.1	OVCAR-3	2267	99.4
1667	3959	61.7	A2780	1750	99.2
736	3939	51	CEM	19	1.3
1689	3697	81.1	Normal controls		
1535	2926	48.5	IOSE-4	1687	100
1503	2401	82.1	IOSE-6	1668	100
1580	1866	67.1	398	1382	94
1592	1530	71.7	1744	1232	42
1637	1365	64.3			

HER2 is expressed in the cell surface of all ascites and solid tumor-derived tumor cells. HER2 expression was assessed using flow cytometry in a large panel of clinical ascites and solid tumor specimens. A breast cancer cell line (MDA 468) and a leukemia cell line (CEM) which do not express any HER2 were used as negative controls. HER2 was found to be expressed in all tested ascites and solid tumor specimens albeit at different levels. HER2 cell surface levels are expressed as specific MFI.

### Flow Cytometry

Mouse anti-human CD3, CD4, CD8, CD45, CD69, CD107a and CD107b mAbs (*BD Biosciences)* were used for phenotypic analysis. 7-AAD was used for viability staining. HER2 surface expression was evaluated using biotin-conjugated anti-HER2 affibody (Abcam) followed by PE-labeled streptavidin. Anti-HER2 CAR surface expression was evaluated using recombinant human HER2 Fc chimera followed by PE-conjugated anti-huIgG. Acquisition and analysis was performed using a BD FACS CANTO II with DIVA software.

**Figure 4:**
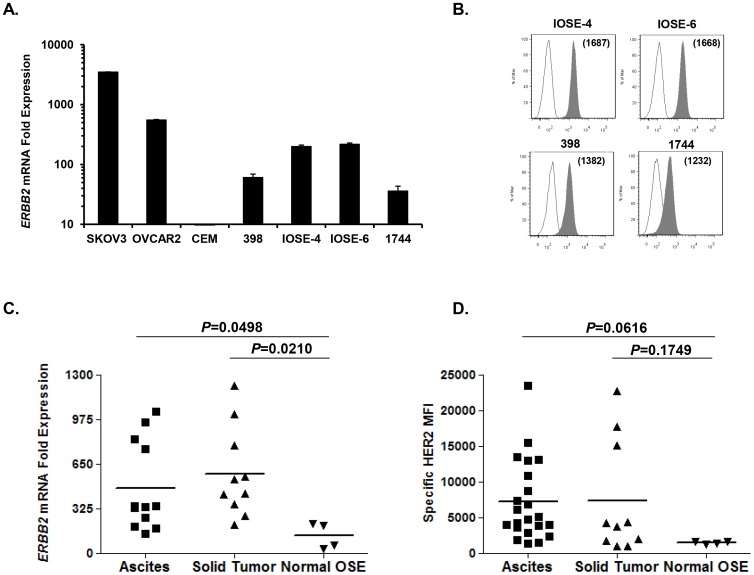
Normal established and primary ovarian surface epithelial cells (OSE) express HER2 protein and *ERBB2* mRNA. ***A.*** *ERBB2* mRNA quantitation in normal OSE cells (398, IOSE-4, IOSE-6 and 1744) by q-PCR. SKOV-3 and CEM were used as positive and negative HER2 expressing cell lines respectively. Bars show the mean ± SD value of triplicate wells. ***B.*** Surface HER2 protein expression (solid histograms) by normal ovarian epithelial cells by flow cytometry; isotype antibody control (open histograms). ***C–D.*** Comparison of the *ERBB2* mRNA and protein levels between ovarian cancer ascites, solid tumor and normal OSE cells. (***C***) Vertical scatter plots of the *ERBB2* mRNA in ascites, solid tumor and normal OSE determined by q-PCR. The mean of each group is indicated by the horizontal line. *P* = 0.0498 when comparing *ERBB2* mRNA in ascites vs normal OSE cells; *P* = 0.0210 when comparing *ERBB2* mRNA in solid tumors vs normal OSE cells. (***D***) Vertical scatter plots of the protein levels in ascites, solid tumor and normal OSE determined by flow cytometry. The mean of each group is indicated by the horizontal line *P* = 0.0616 when comparing HER2 protein in ascites vs normal OSE cells; *P* = 0.1749 when comparing HER2 protein in solid tumors vs normal OSE cells. *P* values were calculated using unpaired student’s t-test analysis.

### Western Blotting

Cell monolayers were washed with phosphate-buffered saline (PBS) and lysed in RIPA buffer (50 mM Tris-HCl (pH 7.0), 1.0% NP-40, 0.1% deoxycholic acid, 30 mM Na_3_VO_4_, 1 mM PMSF). Lysates were cleared by centrifugation and quantified using a Nanoorange Kit (Invitrogen). 15 ug total protein per lane was resolved by sodium dodecyl polyacrylamide gel electrophoresis (SDS–PAGE) in pre-cast gradient (4–15%) gels (Bio-Rad) at 120V for 60 minutes. Protein was transferred from gel to Immobilon-P transfer membrane for 30 minutes, blocked overnight with 5% milk/PBST and blotted using 1 ug/ml of mouse anti-human HER2 mAb (clone 3B5, BD biosciences). Membranes were washed 3× with PBST and blotted with HRP-conjugated anti-mouse secondary antibody for 1 h at room temperature. B-actin was detected using anti-human B-actin-HRP (1∶30,000). Membranes were incubated with ECL Plus (GE Healthcare) for 5 minutes and exposed to films for 15–30 sec.

**Figure 5:**
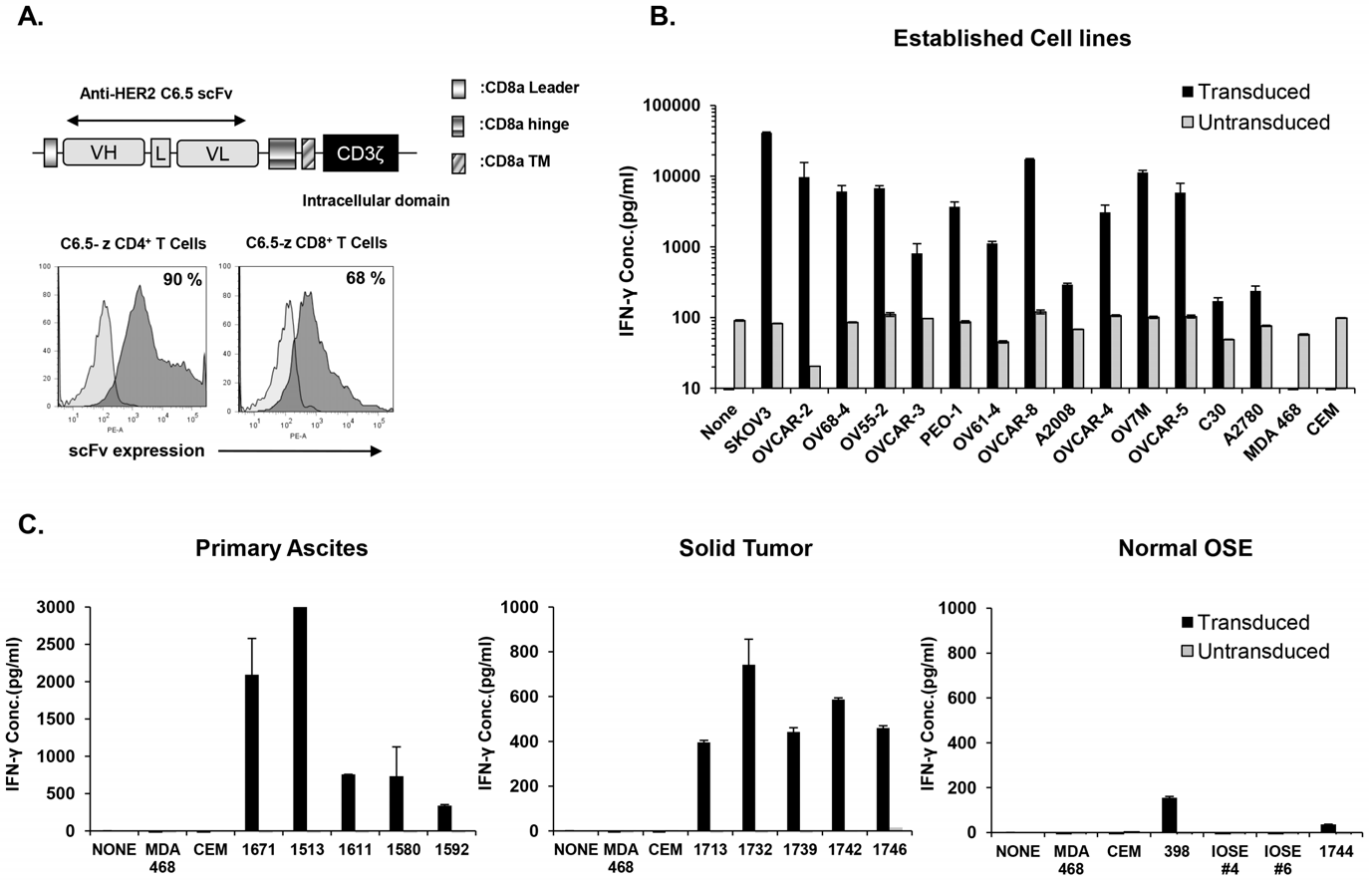
Anti-HER2 CAR-transduced T cells recognize all primary and established ovarian cancer cells. ***A*** **.** Schematic representation of Anti-HER2 Chimeric Antigen Receptor (CAR) construct containing the CD3ζ cytosolic domain alone (C6.5-z). C6.5, anti-HER2 scFv; VL, variable light chain; L, Linker; VH, variable heavy chain; TM, transmembrane region. C6.5 scFv CAR expression (gray histograms) was detected on human CD4^+^ and CD8^+^-gated T cells using recombinant human HER2-Fc chimeric protein 10 days after transduction, compared to untransduced T cells (open histograms). Percentage of CAR transduction is indicated. ***B.*** Anti-HER2 CAR transduced T cells produce IFN-γ specifically after stimulation with human ovarian cancer cell lines. CAR transduced or untransduced T cells were cultured alone (none) or stimulated overnight with human HER2^+^ established and short-term ovarian cancer cell lines or HER2^−^ control lines CEM and MDA468. IFN-γ was quantified from cell-free supernatants by ELISA. ***C.*** Anti-HER2 CAR T cells secrete IFN-γ after stimulation by HER2^+^ primary ascites (left) or solid ovarian (middle) tumor cells. Minimal amounts of IFN-γ were detected after stimulation with the normal ovarian surface epithelial cells 398, IOSE-4, IOSE-6 or 1744 (right). Cytokine concentrations (pg/ml) are reported as the mean ± SEM of triplicate wells.

### Anti-HER2 CAR Construction

PCR products containing the C6.5Y100KA HER2 scFv [20] were kindly provided by Silvana Canevari (Instituto Nazionale dei Tumori, Italy) and then cloned into the pCR2.1-TOPO vector using the Topo TA Cloning Kit (Invitrogen). The C6.5 scFv [21] DNA sequence was developed from the above plasmid using QuikChange Multi Site-directed Mutagenesis Kit (Stratagene). The final plasmid was used as a template for PCR amplification of a 795-bp C6.5 scFv fragment using the following primers: 5′-GCGGGATCCATGGCCCAGGTGCAGCTGTTGCAGTCTGGGGCA-3′ (BamHI is underlined) and 5′-GCGGCTAGCCGCACCTAGGACGGTCAGCTTGGTCCCTCCGCC-3′ (NheI is underlined). The resulting PCR product was digested with BamHI and NheI and ligated into the third generation self-inactivating lentiviral expression vector pCLPS containing a CD3ζ signaling CAR sequence, with transgene expression driven by the CMV promoter. The resulting construct was designated pCLPS-C6.5-z.

**Figure 6:**
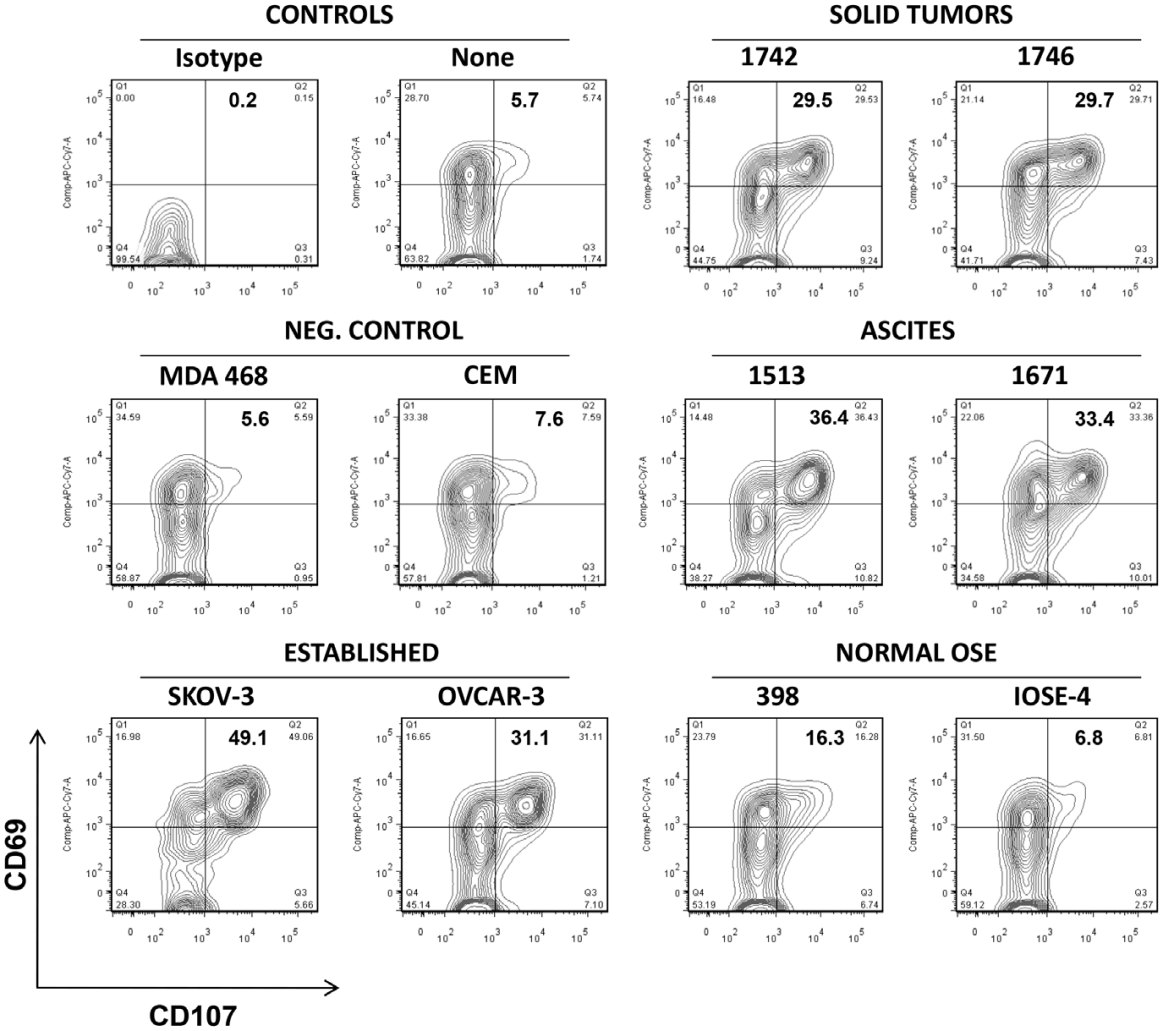
Ovarian cancer cells stimulate a cytolytic phenotype of anti-HER2 CAR T cells. C6.5 CAR T cells degranulate and express T cell activation markers in response to HER2-specific stimulation. C6.5 CAR T cells were cultured without target cells (none) or with the indicated HER2-negative or -positive established and primary tumor cell targets or normal OSE cells for 5 h while being stained by an anti-CD107a, b antibody conjugated with FITC. After the incubation period, T cells were stained for CD8 and CD69 and analyzed by flow cytometry.

### Recombinant Lentivirus Production

High-titer replication-defective lentiviral vectors were produced and concentrated as previously described [22]. Briefly, 293T cells were transfected with 7 ug pVSV-G plasmid, 18 ug pRSV.REV plasmid, 18 ug pMDLg/p.RRE plasmid, and 15 ug pCLPS-C6.5-z transfer plasmid using Express In (Open Biosytems). Viral supernatant was harvested at 24 h and 48 h post-transfection. Viral particles were concentrated by ultracentrifugation for 3 h at 25,000 rpm with a Beckman SW28 rotor (Beckman Coulter) and resuspended in 0.4 ml RPMI.

### T Cell Transduction

Primary human T cells purchased from the Human Immunology Core at University of Pennsylvania were isolated from healthy volunteer donors following leukapheresis by negative selection. All specimens were collected under a University Institutional Review Board-approved protocol, and written informed consent was obtained from each donor. T cells were stimulated in complete media with anti-CD3 and anti-CD28 mAb coated beads (Invitrogen) and transduced with recombinant CAR-encoding lentivirus at MOI of ∼5–10 as described [22].

### Cytokine Release Assays

1×10^5^ T cells were co-cultured with 1×10^5^ target cells per well in triplicate in 96-well round-bottom plates in a final volume of 200 ul of complete media. After 20∼24 hr, cell-free supernatants were assayed for presence of IFN-γ using either an ELISA Kit (Biolegend) or Cytokine Bead Array (BD Biosciences), according to manufacturers’ instructions.

### Degranulation Assay

Assay was performed as described [23] with minor modifications. 1×10^5^ T cells were co-cultured with 1×10^5^ target cells in 100 ul media per well in a 96-well plate in triplicate. Control cultures contained T cells alone. Anti-CD107a and Anti-CD107b Ab (10 ul/well) or IgG1 conjugated to FITC (BD Biosciences), and 1 ul/sample of monensin (BD Biosciences) were added to culture and incubated for 5 h at 37°C. Cells were washed twice with PBS, stained for expression of CAR, CD8 and CD69 and analyzed by flow cytometry as described above.

### Chromium Release Assay

^51^Cr release assays were performed as described [24]. Target cells were labeled with 100 uCi ^51^Cr at 37°C for 1.5 hours. Target cells were washed three times in PBS, resuspended in culture medium at 1×10^5^ viable cells/ml and 100 ul added per well of a 96-well V-bottom plate. Effector cells were washed twice in culture medium and added to wells at the given ratios. Plates were quickly centrifuged to settle cells, and incubated at 37°C in a 5% CO_2_ incubator for 18 hours after which time the supernatants were harvested, transferred to a lumar-plate (Packard) and counted using a 1450 Microbeta Liquid Scintillation Counter (Perkin-Elmer). Spontaneous ^51^Cr release was evaluated in target cells incubated with medium alone. Maximal ^51^Cr release was measured in target cells incubated with SDS at a final concentration of 2% (v/v). Percent specific lysis was calculated as (experimental - spontaneous lysis/maximal - spontaneous lysis) times 100.

### Statistical Analysis

GraphPad Prism 4.0 (GraphPad Software) was used for the statistical calculations. *P*<0.05 values were considered significant. R-squared values (R^2^) were calculated using linear regression via Microsoft Excel.

## Results

### Immunohistochemical Detection of HER2 Expression in Ovarian Cancer

HER2 expression was evaluated in 50 cases of high-grade ovarian serous carcinomas using immunohistochemical (IHC) analysis in a tissue microarray (TMA). Individual cases varied with respect to the number of tumor sites available for analysis. All cases contained at least one primary lesion site while the majority of cases (96%) had both primary and ≥1 metastatic site available. For many samples, there were ≥2 (62%) or ≥3 (42%) metastatic sites available on the TMA. According to IHC scoring, HER2 was expressed at various levels among the 50 cases ranging from undetectable (score 0) to strong staining (score 3+; **Figure 1A**). Positive HER2 expression at any level was detected in 26 cases (52%) while no HER2 was detectable at any site in 24 cases (**Figure 1B**). Out of the 26 cases expressing HER2 at one or more sites, only 6 (23%) expressed HER2 at all sites by IHC; such cases were weighted toward higher HER2 expression. Most cases that expressed HER2 at any site (20/26) exhibited discordant expression (detectable versus undetectable) at one or more tumor sites. The mean number of evaluated sites was similar amongst the 26 cases with detectable HER2 and the 24 cases with no detectable HER2 (4.2 vs. 3.7, respectively; culture medium *P* = 0.19). Heterogeneity in the patterning of HER2 protein expression among the different sites in individual cases was present with multiple patterns: detectable in all (6/50); detectable in primary but undetectable in metastatic tumor (8/50); detectable in metastatic but undetectable in primary tumor (5/50); detectable in at least one primary and one metastatic sites (7/50). Of 195 tumor sites evaluated, 138 (71%) showed no detectable HER2 expression (**Figure 1C**). Fifty-seven (29%) had positive HER2 expression; 25% (49/195) with a >0≤1 HER2 score; 2.5% (5/195) with a >1≤2 HER2 score; and 1.5% (3/195) with a >2≤3 HER2 score. A greater frequency of primary tumor sites expressed HER2 (36%; 30/84) compared to metastatic sites (24%; 27/111) and had a higher mean HER2 expression score (0.37 *vs.* 0.21, *P* = 0.04; **Figure 1D**). Among primary and metastatic sites that expressed HER2 at any level, there was no statistically significant difference in expression level (1.03 *vs.* 0.86, *P* = 0.26). In conclusion, IHC allows for the detection of HER2 expression in 29% of all sites and approximately half of all advanced OvCa cases tested.

### HER2 is Expressed by All Ovarian Cancer Cell Lines

The relatively low sensitivity of IHC analysis can influence the detection of low abundance proteins and therefore misrepresent the true frequency of cancers that express HER2. To better evaluate HER2 expression in OvCa, 12 established and 7 short-term OvCa cell lines were measured for *ERBB2* mRNA levels by q-PCR, and compared to that detected from CEM, a human T cell lymphoblast-like cell line that lacks HER2 expression [25]. All OvCa cell lines expressed *ERBB2* mRNA, albeit at various levels (**Figure 2A**). Increased *ERBB2* mRNA expression, relative to the CEM control, ranged from 63- to 6614-fold in established lines and from 40- to 1088-fold in short-term primary cell lines. Mean relative *ERBB2* mRNA expression amongst established (815-fold) and short-term OvCa cell lines (372-fold) was not statistically different from one another (*P* = 0.45). *ERBB2* mRNA was not detected in the CEM control line, but was detectable at low levels in the control breast cancer line, MDA468, which expresses *ERBB2* mRNA but not surface HER2 protein [25].

HER2 protein expression was examined on established and short-term primary tumor cell lines by staining non-permeabilized cells with a highly sensitive anti-HER2 affibody [25], a high affinity ligand against the extracellular domain of HER2, followed by flow cytometric analysis. Representative histograms show established OvCa cell lines expressing high, intermediate or low surface levels of HER2 protein, and negative control cell lines with no detectable HER2 expression (**Figure 2B**). All established (n = 12) and short-term (n = 7) OvCa cell lines tested showed HER2 protein surface expression **(** **Table 1** **)**. Most cells in established (97.7±1.3%) and short-term lines (88.9±4.8%) expressed HER2 protein, with a higher percentage in the established lines (*P* = 0.03) **(** **Table 1** **)**. Mean HER2 protein levels measured by specific mean fluorescence intensity (MFI) trended towards being higher in established cell lines (1906±1221 FIU; fluorescence intensity units) compared to short-term cell lines (594±165 FIU) but were not significantly different (*P* = 0.43) **(** **Table 1** **)**, consistent with mRNA results. HER2 protein and *ERBB2* mRNA expression levels from all OvCa cell lines were significantly correlated (R^2^ = 0.83; calculated using linear regression). Cellular HER2 protein expression was confirmed by western blot analysis (representative samples shown; **Figure 2C**).

### Broad HER2 Expression in Primary Ovarian Cancer Specimens

Since *in vitro* culture may selectively enrich for HER2-expressing OvCa cells [16], we measured HER2 expression in uncultured tumor cells derived directly from primary peritoneal OvCa ascites or freshly resected solid tumors. Tumor cells from ascites or solid tumor specimens were enriched by magnetic depletion of CD45^+^ leukocytes and assessed for relative *ERBB2* mRNA levels via q-PCR (**Figure 3A, B**). All primary uncultured tumor cells tested (n = 22) expressed *ERBB2* mRNA at levels ranging from 35- to 1225-fold higher than CEM cells. No significant difference in mean *ERBB2* mRNA level was observed between ascites and solid tumors (477 *v*s. 583, respectively; *P* = 0.46). Flow cytometry performed using anti-HER2 affibody showed that non-permeabilized tumor cells (BerEp4^+^ CD45^−^) in all primary ascites (n = 22) and solid tumor samples (n = 10) expressed surface HER2 protein, albeit at variable levels, in agreement with the detection of *ERBB2* mRNA by q-PCR **(** **Table 2**; representative data shown in **Figure 3C**). No significant difference in HER2 protein level was observed between ascites or solid tumors derived cells (7209±1197 FIU *vs.* 7407±2531 FIU, respectively; *P* = 0.95), in agreement with mRNA results. Western blot analysis performed on whole tumor lysates also showed ubiquitous expression of HER2 in primary solid tumors (representative samples shown in **Figure 3D** **)**.

### Detectable HER2 Expression in Normal Ovarian Epithelial Cells

Three immortalized ovarian surface epithelium cell lines (OSE) and one primary uncultured cell specimen (1744) derived from normal ovaries were tested for HER2 expression. All normal OSE cells expressed low levels of *ERBB2* mRNA that ranged from 35-fold to 217-fold higher than CEM control cells (128±5.5; **Figure 4A**). Flow cytometry (**Figure 4B**) and western blot analysis (not shown) indicated that all normal OSE cells express low yet detectable levels of HER2 protein. IOSE-6 and IOSE-4 cell lines expressed higher protein levels than the 398 line and 1744 normal ovary specimen, in accordance with mRNA results.

Next we compared the *ERBB2* mRNA and protein levels in normal OSE cells, malignant primary ascites and solid tumor-derived tumor cells (**Figures 4C–D**). Ascites and solid tumor samples generally expressed higher levels of *ERBB2* mRNA and protein compared to OSE cells, though an overlap in expression level did exist among groups. Ascites and solid tumors expressed significantly more *ERBB2* mRNA than OSE cells (Ascites, 483±319-fold, *P* = 0.0498; Solid tumor, 583±330-fold, *P* = 0.0210; OSE, 128±93-fold). HER2 protein levels tended to be higher in ascites (5825±1202-fold) and solid tumor samples (7407±2531-fold) compared to normal OSE (1429±111-fold), but to a low statistical significance (Ascites *v*s. OSE *P* = 0.0616; Solid *vs.* OSE *P* = 0.1749). Variation in HER2 expression levels in OSE samples was limited, allowing a reliable discrimination of tumor samples that overexpress HER2. By comparison, 75% (9/12) of ascites and 90% (9/10) solid tumors expressed higher levels of HER2 compared to OSE, and 91% (20/22) of ascites and 80% (8/10) solid tumors had higher HER2 protein levels than normal OSE cells.

### HER2-specific T Cells Recognize All Ovarian Carcinoma Cell Lines

Chimeric antigen receptors (CARs) combine antibody specificity for a surface antigen with the effector activity of T lymphocytes [26]. HER2-specific CAR-expressing T cells can exert potent, dose-dependent *in vitro* anti-tumor activity against HER2-positive target cells [20], [25]. To investigate the extent to which broad HER2 expression by OvCa confers sensitivity to HER2-targeted immunotherapy, human donor T cells were genetically modified to express an anti-HER2 CAR (C6.5) [27], thereby redirecting them against surface HER2, and tested for specific activity against OvCas. The C6.5 CAR construct is comprised of the C6.5 scFv linked to a CD8α hinge and transmembrane region, followed by an intracellular CD3ζ signaling domain (C6.5-z; **Figure 5A**). Primary human CD4^+^ and CD8^+^ T cells were efficiently transduced with CAR using lentiviral vectors with transduction efficiencies reproducibly >60% (**Figure 5A**). In co-culture, anti-HER2 CAR T cells recognized and responded to stimulation by all established (n = 10) or short-term (n = 4) human OvCa cells, but not against CEM and MDA-468 cell lines lacking HER2 expression (**Figure 5B**). The amount of IFN-γ secreted was associated with the level of HER2 protein expressed by tumor cells (R^2^ = 0.8064; calculated using linear regression). Untransduced T cells did not produce cytokines upon tumor stimulation, demonstrating the requirement for antigen-specificity. This was confirmed using control anti-mesothelin CAR transduced T cells [22] which did not recognize tumors that express HER2 but lack mesothelin (**Figure S1**).

Importantly, anti-HER2 CAR T cells were also capable of specific recognition and IFN-γ secretion upon stimulation with Ber-Ep4-enriched, primary cancer cells derived from any ascites (n = 5) or solid tumor tested (n = 5; **Figure 5C**). In contrast, anti-HER2 CAR T cells secreted little to no significant amounts of IFN-γ when cultured with normal OSE cells expressing low levels of HER2 antigen (**Figure 5C**). Anti-HER2 CAR T cell effector function was confirmed by monitoring degranulation as a quantitative indicator of T cell cytotoxic activity [23]. Anti-HER2 CAR-expressing T cells exhibited a cytolytic phenotype in response to all established and primary HER2^+^ OvCa cells, while minimal degranulation was observed against normal OSE cells (**Figure 6**). Furthermore C6.5 CAR T cells directly and efficiently lysed representative HER2^+^ human established ovarian cancer cells (OVCAR-3) and primary human solid tumor (1513) or ascites (1742) cancer cells during an 18 hour chromium release assay. C6.5 CAR T cells did not lyse HER2^−^ tumor cell lines or normal IOSE-4 cells **(Figure S2)**.

## Discussion

*ERBB2* gene amplification and/or overexpression has been reported in a subset of OvCas [6], [7], [10], [11], [12], [13], [14], [15] and is associated with poor clinical outcome [10], [28]. In these studies, detection relied largely upon IHC analysis to monitor protein over-expression or fluorescence *in situ* hybridization (FISH) to assess gene amplification. In IHC, the amount of protein on the cell surface is determined in a semi-quantitative manner. Samples are scored in a four-tiered scoring system from 0 to 3+ [9]. In FISH, *ERBB2* gene copy number is determined using locus-specific DNA probes [29]. IHC results correlate with FISH results for score 1+ and 3+ samples, but only a limited correlation has been reported for score 2+ samples [30]. Based on these analyses, a subset of patients with clearly positive HER2-expressing tumor may be eligible for HER2-directed treatment using Herceptin. Although Herceptin can mobilize antitumor immune mechanisms, it functions primarily by disrupting HER2 signaling, and therefore its therapeutic efficacy requires overexpression and constitutive activation of HER2 [31]. However, alternate HER2-targeted therapies may be effective even at low levels of surface HER2 expression.

In our study, positive *ERBB2* mRNA and protein expression was found in all established and short-term cultured OvCa cell lines tested as well as in fresh tumor cells derived from ascites or solid tumor utilizing RT-PCR, flow cytometry and western blot analyses. Detection of ubiquitous expression may be explained by the use of these techniques, which are more sensitive than IHC and FISH for measuring HER2 protein and *ERBB2* mRNA levels [32], [33]. Indeed, IHC is often applied on fixed tissue where the fixation methods may adversely influence the specificity and sensitivity of the applied antibodies [34]. HER2 testing via IHC on frozen material is less prone to false results but frozen material is rarely available [35], [36]. In contrast, surface HER2 protein expression can be detected on cells with high sensitivity via flow cytometry [16]. Despite its merits, FISH-based detection does not assess gene expression and cannot identify gene product overexpression in the absence of gene amplification. However, *ERBB2* mRNA expression can be measured via q-PCR, a fast, reliable and cost-effective alternative to the combination of IHC and FISH procedures, and also correlates with overall and disease-free survival [37].

Our results expand upon those reported by Hellstrom et al. [16], where broad HER2 expression was observed principally on in vitro cultured cell lines established from stage II and IV OvCa via flow cytometry. Binding of the Herceptin antibody inhibited [^3^H]-thymidine incorporation by two established primary OvCa cell lines, and facilitated antibody-dependent cell-mediated cytotoxicity (ADCC) by allogeneic immune cells against one cell line [16]. This study was limited by the postulated enhancement of HER2 expression via extended cell culture, use of a single methodology for expression analysis against the established cell panel, and limited numbers of samples tested for HER2-specific immune recognition by Herceptin. In our study, we show positive HER2 expression in both established and short-term cultured lines at both the mRNA and protein level via three independent techniques. Strikingly, broad HER2 expression by ovarian cancer cells is not restricted to established cell lines but was found in all fresh solid tumor and ascites-derived cancer cells in the absence of extended cell culture or manipulation. HER2 expression was generally higher than that detected in normal OSE, suggesting a window of opportunity for cancer targeting. Accordingly, HER2-redirected T cell responses, including proinflammatory cytokine secretion and degranulation, were detected following stimulation with a wide variety of established tumor cells or fresh resected solid tumor or ascites samples, indicating the HER2 is expressed by all OvCa samples at an immunologically active level. Hence, immune recognition was not a phenomenon observed exclusively against established OvCa cell lines which might have undergone *in vitro* selection for high HER2 expression. Lastly, immune recognition against immortalized or primary normal OSE cells was limited or absent, consistent with lower HER2 expression by these cells.

The anti-HER2 CAR used in our study contains the C6.5 scFv with an affinity (1.6×10^−8^M) above the threshold required to confer reactivity to primary T cells against HER2-expressing tumor cells [27]. Others have shown that T cells bearing a lower affinity C6.5-derivative CAR (C6.5G98A; Kd>10^−8^M) are exclusively activated by high HER2-expressing tumor cells, and that C6.5-derived CAR T cells of any affinity had no reactivity against cells from normal tissues [27]. In our study, C6.5 CAR-grafted T cells reacted against all established or primary OvCa cells and exhibited minimal or no reactivity against normal OSE, which generally express lower levels of HER2 than malignant ovarian cells. In another study, Zhao and coworkers, constructed an anti-HER2 CAR comprised of a Herceptin-derived scFv (4D5), where the affinity of the scFv (3×10^−10^ M) is approximately two orders of magnitude higher than that of the C6.5 scFv and recognizes a different epitope [25], [38]. 4D5 CAR T cells reacted against a panel of tumor cells of different origin expressing even low levels of HER2, as well as multiple normal cells [25], [39]. Administration of high numbers of T cells expressing the 4D5 CAR encompassing CD28 and CD137 costimulatory domains following lymphodepletion resulted in an assumed response against lung epithelial cells expressing low levels of HER2, leading to cytokine storm, respiratory distress and ultimately patient death [39]. The impact of high anti-HER2 CAR affinity and epitope specificity on immune recognition and response to normal cells in patients, resulting in the development of toxicity, remains unresolved.

The breadth of HER2 expression among OvCas has a direct implication on determining patients’ access to various HER2-targeting therapeutic strategies and drug delivery systems. In contrast to breast cancer, trastuzumab (Herceptin) targeting of HER2 mediated limited responses (7%) in patients with advanced OvCa [12] despite the paradoxical finding of ubiquitous HER2 expression. The mechanisms accounting for the poor response to trastuzumab in OvCa patients remain unknown. Resistance of tumors to trastuzumab might arise through alternative signaling and survival pathways induced as a consequence of a marked inhibition of HER2 [40]. Alternatively, since the activity of trastuzumab is partially mediated through ADCC and is dependent upon a functional immune system [41], it remains possible that administration of chemotherapy prior to trastuzumab treatment, as performed above [12], may hamper overall immune function and ADCC activity. Further complicating effective therapy is the finding that ovarian cancers are prone to harbor an immunosuppressive microenvironment with a limited effective innate compartment necessary for mediating ADCC [42]. HER2-specific peptide and DNA vaccines that induce T cell responses against HER2 represent alternative treatment strategies, however these approaches have not evolved beyond phase I/II studies due to limited clinical efficacy [43], [44], [45]. However, T cells engineered to express a HER2-specific CAR have the capacity to recognize HER2 on the surface of all ovarian cancer cells, and thus represent an attractive option for therapy. Despite their potential for toxicity [39], CARs against HER2 have been well characterized, have high *in vitro* and *in vivo* potential, and remain under clinical investigation [27], [46], [47], [48], [49].

We conclude that HER2 is broadly expressed in established and primary ovarian carcinomas. Despite the wide range of HER2 expression, all OvCas were sensitive to recognition by genetically modified T cells bearing an anti-HER2 CAR indicating that the surface expression of HER2 is sufficient for the induction of potent immune responses. We postulate that HER2-directed immunotherapeutic strategies may have beneficial effects in patients with ovarian carcinoma, including many of those who are negative according to routinely applied immunohistochemistry on tumors resected at primary surgery. However, careful design and testing is needed to understand how to safely apply HER2 targeted approaches for the treatment of patients with HER2-expressing ovarian carcinomas.

## Supporting Information

Figure S1 **Specific HER2 CAR-redirected recognition of HER2-expressing tumor cells.** C6.5-z anti-HER2 or P4-z anti-mesothelin CAR T cells were co-cultured with tumor cells expressing both HER2 and mesothelin or only HER2 or lacking both antigens. Cell-free supernatant from three independent cultures was harvested and pooled after ∼20 hours of incubation and the IFN-γ secretion was quantified using cytometric bead array technology. Values represent cytokine concentration (pg/ml).
(TIF)

Figure S2 **Direct lytic activity of anti-HER2 lentiviral vector-engineered T cells.** Antigen-specific killing of HER2^+^ tumor cells by C6.5 CAR T cells. Primary human T cells transduced to express the C6.5 CAR (∼30% expression) were co-cultured with ^51^Cr-labeled HER2 positive or negative tumor cells or normal OSE for 18 hrs at the indicated effector to target ratio. Percent specific target cell lysis was calculated as (experimental - spontaneous release) ÷ (maximal - spontaneous release)×100. Results are graphed with respect to effective E/T ratio and represent mean (±SEM) cytotoxicity of triplicate wells.
(TIF)
