# Supplementary figures and images for: Primary Human Ovarian Epithelial Cancer Cells Broadly Express HER2 at Immunologically-Detectable Levels
Source: PLoS One. 2012 Nov 26;7(11):e49829. doi: 10.1371/journal.pone.0049829 (PMC3506636; doi:10.1371/journal.pone.0049829)

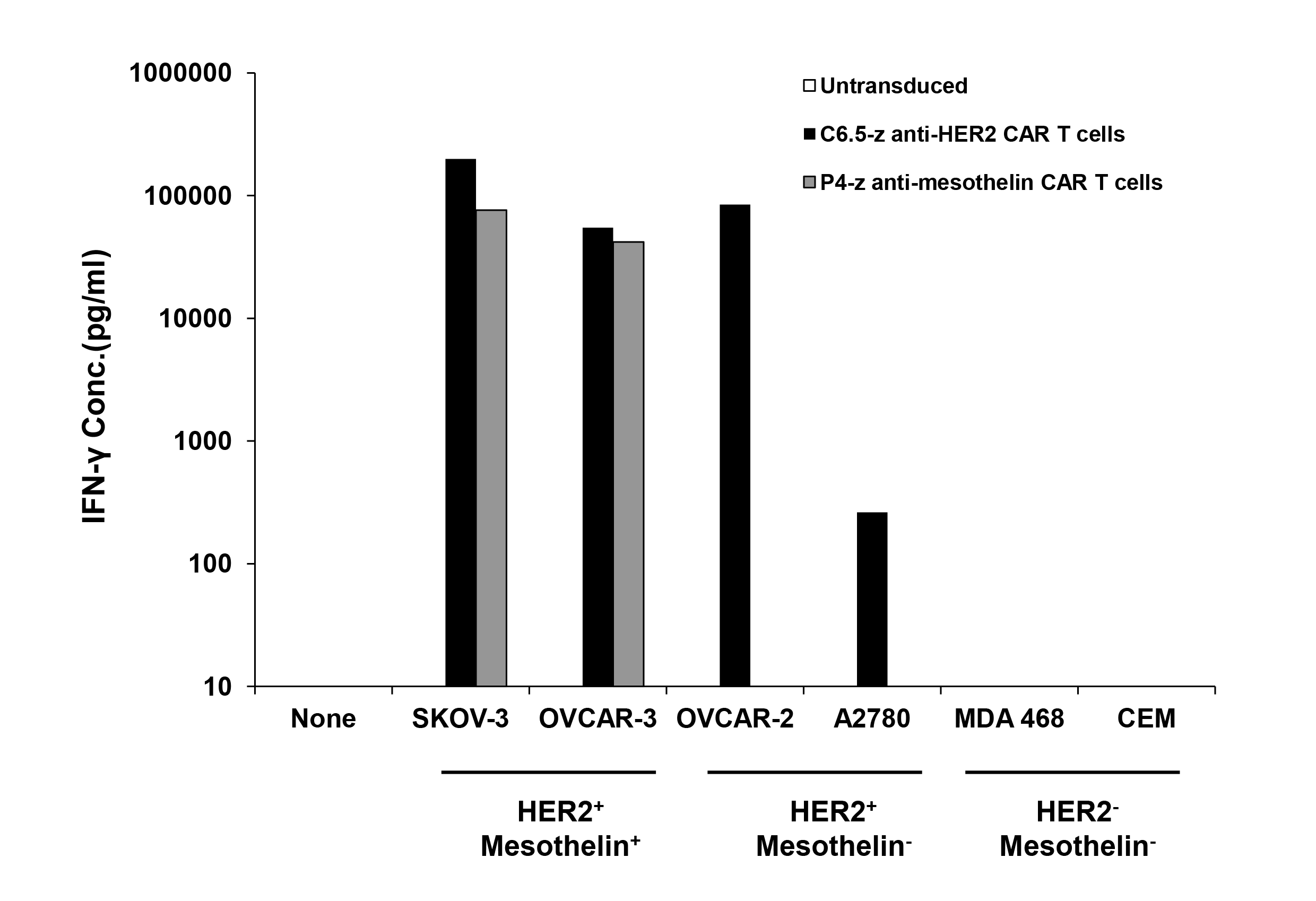

Supplement: Figure S1 — Specific HER2 CAR-redirected recognition of HER2-expressing tumor cells. C6.5-z anti-HER2 or P4-z anti-mesothelin CAR T cells were co-cultured with tumor cells expressing both HER2 and mesothelin or only HER2 or lacking both antigens. Cell-free supernatant from three independent cultures was harvested and pooled after ∼20 hours of incubation and the IFN-γ secretion was quantified using cytometric bead array technology. Values represent cytokine concentration (pg/ml). (TIF) [file pone.0049829.s001.tif]

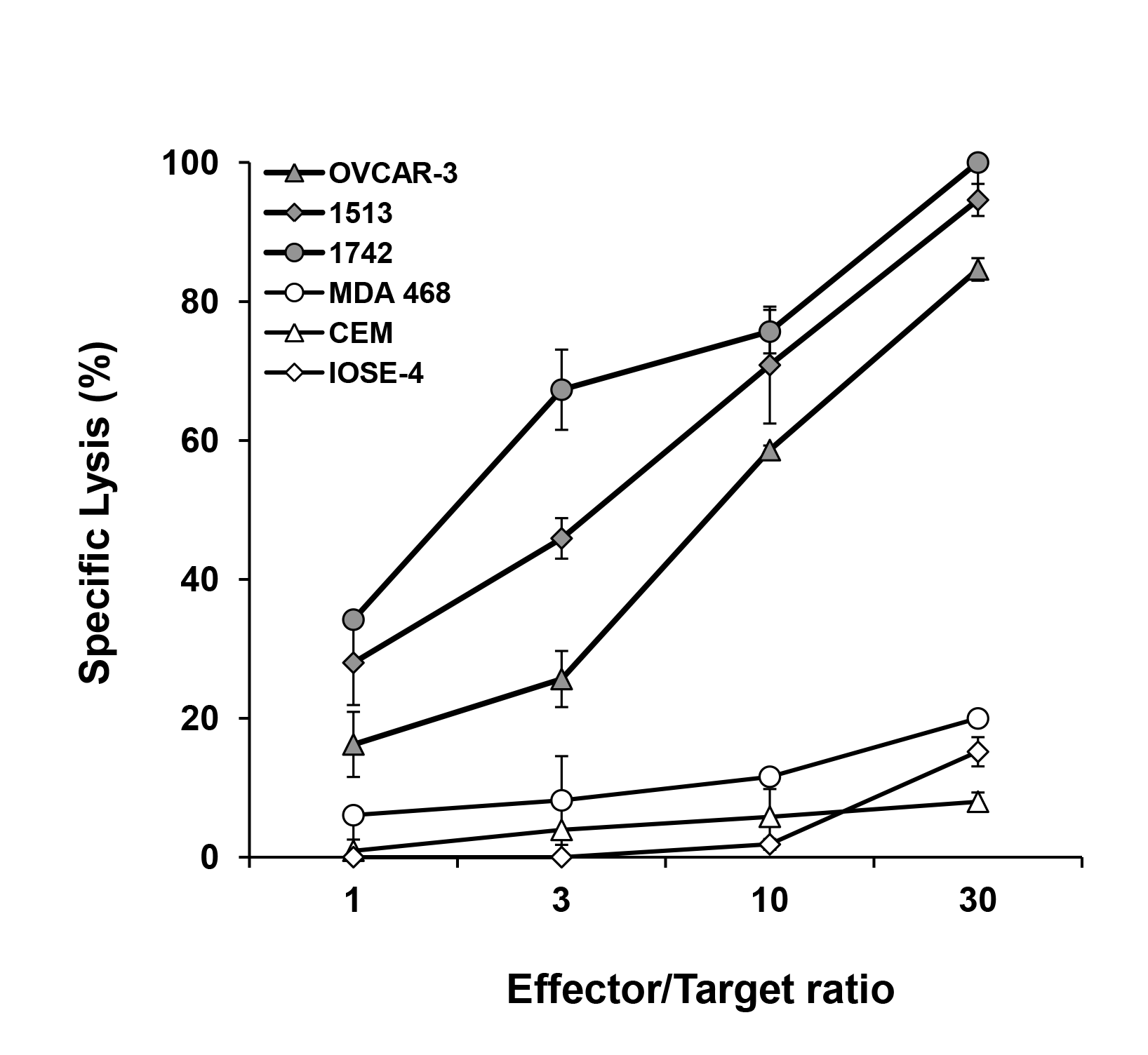

Supplement: Figure S2 — Direct lytic activity of anti-HER2 lentiviral vector-engineered T cells. Antigen-specific killing of HER2+ tumor cells by C6.5 CAR T cells. Primary human T cells transduced to express the C6.5 CAR (∼30% expression) were co-cultured with 51Cr-labeled HER2 positive or negative tumor cells or normal OSE for 18 hrs at the indicated effector to target ratio. Percent specific target cell lysis was calculated as (experimental - spontaneous release) ÷ (maximal - spontaneous release)×100. Results are graphed with respect to effective E/T ratio and represent mean (±SEM) cytotoxicity of triplicate wells. (TIF) [file pone.0049829.s002.tif]
